# Acute Tonsilitis

**Published:** 1893-03

**Authors:** 


ACUTE TONSILITIS.—Professor Cohen gave the following formula for a gargle, which he considers one of the very best for acute tonsillitis :

℞ Tr. guaiac. ammoniat.,

Tr. cinchonæ comp., aa f℥ij.

Mel despumati, . . . . . . . . ℥vj.

And,

℞ Potassii chloridi,... . . . . . ℥ij.

Aquæ medth. piperit.,. . f ℥iv.

M. Use as a gargle.

The manner of making the above preparations is as follows . Dissolve the potassii chloridum in the peppermint-water; put the honey in a bottle, and, by shaking, get it thoroughly spread over the sides ; add the guaiac and cinchona, and shake the bottle while doing so, in order that they may get well mixed with the honey ; then add the potassii chloridum solution and again shake the bottle thoroughly. The manner of making this has much to do with whether it will be pleasant or not. If it is prepared as above it will be a very agreeable preparation.—*Ex.*

